# A Graphene-Based Stopband FSS with Suppressed Mutual Coupling in Dielectric Resonator Antennas

**DOI:** 10.3390/ma14061490

**Published:** 2021-03-18

**Authors:** Wei Qian, Wei Xia, Wenqing Zhou, Rongguo Song, Xin Zhao, Daping He

**Affiliations:** 1 Hubei Engineering Research Center of RF-Microwave Technology and Application, Wuhan University of Technology, Wuhan 430070, China; qianwei@whut.edu.cn (W.Q.); dxk_xiawei@whut.edu.cn (W.X.); rongguo_song@whut.edu.cn (R.S.); xzhao@whut.edu.cn (X.Z.); 2 School of Electronic Science and Engineering, Hunan University of Information Technology, Changsha 410138, China; wenqing0508@whut.edu.cn

**Keywords:** mutual coupling reduction, dielectric resonator antenna array, frequency-selective surface, high-conductivity graphene films

## Abstract

A novel stopband frequency-selective surface (FSS) made of high-conductivity graphene assemble films (HCGFs) for reducing the mutual coupling between dielectric resonator antennas (DRAs) is investigated and presented. The FSS is a “Hamburg” structure consisting of a two-layer HCGF and a one-layer dielectric substrate. A laser-engraving technology is applied to fabricate the FSS. The proposed improved Jerusalem cross FSS, compared with cross FSS and Jerusalem cross FSS, can effectively reduce the size of the unit cell by 88.89%. Moreover, the FSS, composing of 2 × 10-unit cells along the E-plane, is proposed and embedded between two DRAs, which nearly has no effect on the reflection coefficient of the antenna. However, the mutual coupling is reduced by more than 7 dB on average (7.16 dB at 3.4 GHz, 7.42 dB at 3.5 GHz, 7.71 dB at 3.6 GHz) with the FSS. The patterns of the antenna are also measured. Therefore, it is suggested that the proposed FSS is a good candidate to reduce mutual coupling in the multiple-input–multiple-output (MIMO) antenna system for 5G communication.

## 1. Introduction

Recently, there has been an increasing demand for high-capacity and fast rate in the field of communication. The development of multiple-input–multiple-output (MIMO) antennas is vital because it can provide spatial multiplexing gain, diversity gain, and interference reduction capability. In MIMO systems, the inter-element spacing should be minimum for high channel capacity and excellent signal-to-noise ratio. However, the inter-element spacing is usually chosen as half of the wavelength due to implementation limitations, leading to strong mutual coupling between radiating elements. The strong coupling will have an adverse effect on the radiation pattern, return loss, and bandwidth, leading to further performance degradation [1]. The problem of mutual coupling among the radiating elements is often solved by using defected ground structure (DGS) [2], parasitic elements [3,4], electromagnetic bandgap (EBG) structures [5,6], metamaterial-based resonators [7,8], and frequency-selective surfaces (FSS) [9,10]. Among them, FSS is widely used due to its simple design, easy processing, and excellent effect [11].

FSSs, composed of periodic conductive patches or aperture elements, are designed to reflect, transmit, or absorb electromagnetic (EM) waves. Most of the investigations of FSSs utilized the strong light-matter interactions between EM field and metals by constructing two-dimensional (2D) periodic arrays composed of metallic grids or dipole antennas [12,13]. However, metal materials are questionable at very high-power transmission or strong incident fields [14,15]. Graphene materials, on the other hand, become better alternatives, which can endow the FSS with new features due to their excellent properties of high conductivity, high thermal conversion efficiency, saturable absorption, and stabilization in complicated environments [16]. As far as we know, most investigations of graphene-based FSS are focused on the THz band [17,18,19,20]. A mantle cloaking method making resonating strip dipole antennas “invisible” to each other was proposed to reduce the mutual coupling between antennas by Yakovlev et al. [18,19]. By controlling the temperature for graphene growth, Chen. et al. demonstrated two kinds of microwave absorbers based on multilayered-graphene FSSs [21]. In the work of Xu et al., a tunable absorber was realized using patterned graphene metasurface to adjust surface resistance [22]. These studies achieved significant progress for the device applications of graphene. Even though graphene-based FSS has been experimentally realized, the structures of unit cells of FSSs are relatively simple and not accurate enough. Moreover, our group has reported applications of high-conductivity graphene assemble films (HCGFs) in antenna design [23,24]. On this basis, we further study the development of HCGF stopband FSS.

In this paper, a novel stopband FSS made of HCGF is presented. The conductivity of HCGF is up to 1.1 × 10^6^ S/m. As far as we know, it is the first time to realize experimentally such an exact graphene-based FSS using laser-engraving technology. The proposed FSS is simulated, tested, and compared with traditional metal FSS, which shows similar isolation, better return loss, and some specific properties of graphene materials.

## 2. Design and Methods

In general, FSSs are arrays of periodic elements with band-stop or bandpass characteristics [25,26]. When the periodicity of the FSS structure is small compared to the operating wavelength, an equivalent *LC* circuit can be applied to model the structure [27]. Most FSSs are metal patch structures consisting of dielectric substrates and thin metal patches. The inductor represented by *L* results from the conductor strip and the capacitor represented by *C* is from the gap effect between the conductor strips. The surface impedance is equivalent to the impedance of a parallel resonant circuit and the central frequency can be calculated using the equation shown below [28]:

(1) $$Z=\frac{j\omega L}{1-\omega^{2}LC}$$

(2) $$\omega =2\pi \cdot f=\frac{1}{\sqrt{LC}},$$

where *Z* is the impedance of the equivalent *LC* circuit, and *f* is the resonance frequency (3.5 GHz). According to the transmission line theory, the inductance and the capacitance approximation formula of the metal patch unit are as follows [29]:

(3) $$L=-\mu_{0}\frac{D}{2\pi }\log\left(\sin(\frac{\pi w}{2D})\right)$$

(4) $$C=-\varepsilon_{0}\varepsilon_{eff}\frac{2D}{\pi }\log\left(\sin(\frac{\pi s}{2D})\right),$$

where *D*, *w* and *s* are the length, width, and interval of the structure capacitance and inductor, $\mu_{0}$ and $\varepsilon_{0}$ are permeability and permittivity in vacuum, and $\varepsilon_{eff}$ are the effective dielectric constant of the dielectric substrate. $\varepsilon_{eff}$ can be obtained by the following formula:

(5) $$\varepsilon_{eff}=\frac{\varepsilon_{r}+1}{2}+\frac{\varepsilon_{r}-1}{2}\cdot \frac{1}{\sqrt{1+12d/W}},$$

where *d* is the thickness of the substrate, W is the width of the metal patch, and $\varepsilon_{r}$ is the relative dielectric constant of the substrate.

Due to the highly integrated and complicated design of the proposed FSS structure, the equivalent *LC* circuit fails to provide an accurate description thus requiring further optimization. Firstly, a simple structure of traditional cross structure is used for the calculation to obtain the parameters, followed by the simulation to adjust the center frequency and bandwidth. To be more specific, the electrical length and microstrip line width of FSS are calculated by classical theoretical formulas, and then the miniaturization design is carried out by CST simulation software to obtain the optimized structure.

**Figure 1:**
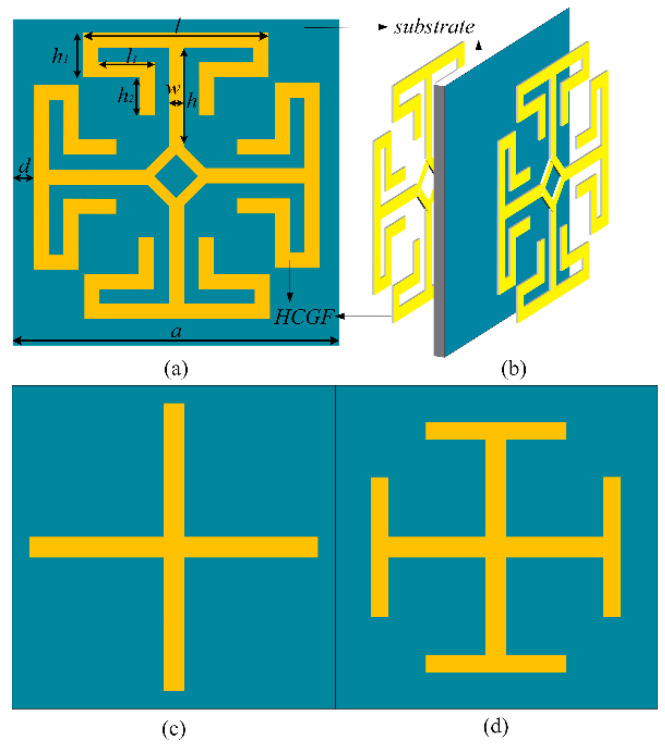
Unit cell configurations. (**a**) Proposed frequency-selective surface (FSS). (**b**) Proposed FSS made by high-conductivity graphene assemble film (HCGF) element configuration. (**c**) Cross FSS. (**d**) Jerusalem cross FSS. *a* = 10 mm, *d* = 0.3 mm, *l* = 7 mm, *h* = 2.7 mm, *w* = 0.3 mm, *h*_1_ = 0.9 mm, *l*_1_ = 2.75 mm, *t* = 0.03 mm.

The proposed stopband FSS made by HCGF element configuration is shown in Figure 1a,b. The proposed FSS is composed of two conventional structures—a cross structure (Figure 1c) and a Jerusalem cross (J-cross) (Figure 1d). The proposed structure has an extended J-cross at each arm end, which can effectively decrease the size of FSS. The size of the proposed FSS, J-cross FSS, cross FSS are 100 mm^2^, 225 mm^2^, and 900 mm^2^, respectively. It is obvious that the proposed FSS structure effectively reduced the size of the unit cell by 88.89%.

**Figure 2:**
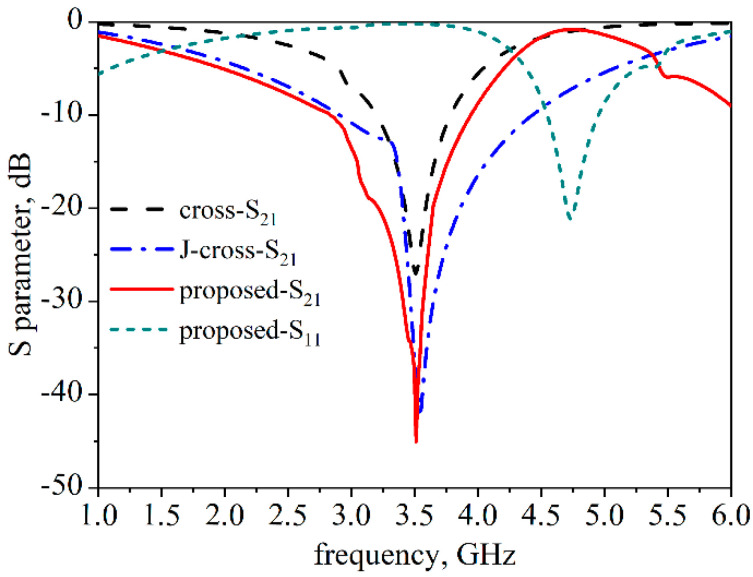
The transmission and reflection characteristics of three different FSS structures.

The proposed structures are printed on both sides of a 1.6-mm-thickFire Resistant-4 (FR4) substrate ($\varepsilon_{r}$ = 4.3, tanδ = 0.025) with a periodicity of 10 mm. The transmission and reflection responses of different structures are depicted in Figure 2. As can be seen, the bandwidths for the traditional cross structure, the classical J-structure, and the proposed structure are 3.20–3.78 GHz, 2.90–4.42 GHz, and 2.93–3.93 GHz, respectively, which can all cover the 3.5 GHz band. In addition, the proposed FSS showed a 41 dB insertion loss at the center frequency, which is better than the other two structures demonstrating its efficiency in reducing mutual coupling.

## 3. Measurement and Results

**Figure 3:**
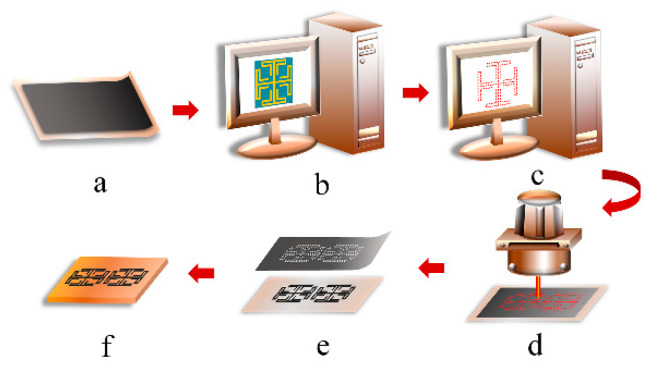
Fabrication scheme of the HCGF FSS. (**a**) Hot pressing of the HCGF onto the polytetrafluoroethylene (PTFE) substrate. (**b**) Exportation of the contour FSS from the simulation tool. (**c**) Importation of the contour file into the laser engraving software for laser path calculation. (**d**) Laser engraving process to cut the path. (**e**) Removal of the unwanted HCGF and detachment of the patterned HCGF from the PTFE substrate. (**f**) Transfer of the patterned HCGF to the FR4 substrate by pressing.

**Figure 4:**
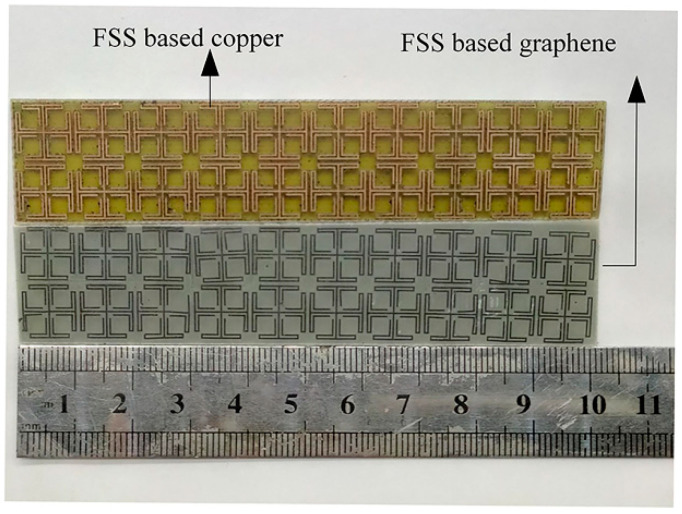
The prototypes of graphene film FSS structure after laser-engraving.

Figure 3a–f illustrated the fabrication scheme of the proposed HCGF FSS structure using the laser-engraving method including the following three steps: firstly, the HCGF with a thickness of 30 μm was attached to polytetrafluoroethylene (PTFE) substrates by hot pressing at 200 °C. Then, the HCGF was subjected to laser engraving to pattern the surface with the designed structure by removing the unwanted part. Finally, the patterned HCGF was transferred to FR4 substrate with a thickness of 1.6 mm for further measurements. The fabricated prototype using the method described above is shown in Figure 4.

**Figure 5:**
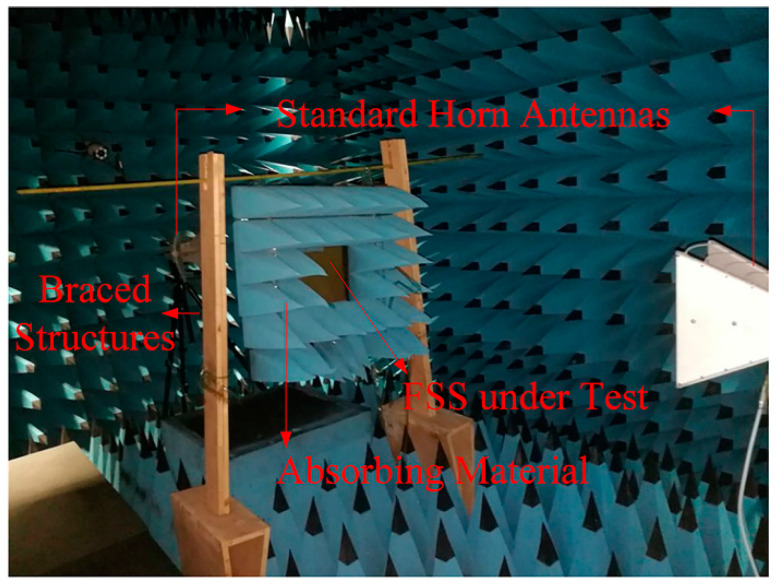
The test environment of FSS under test (FUT).

**Figure 6:**
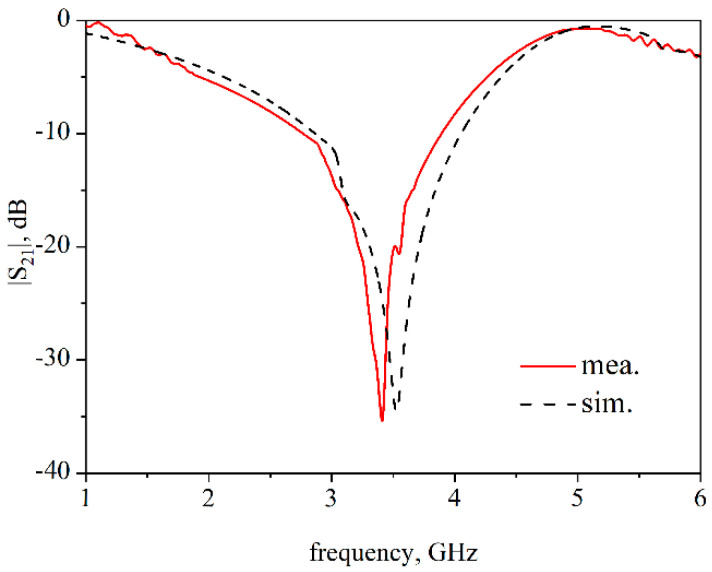
Measured (red line) and simulated (black dashed line) transmission coefficient of FUT.

The performance of the proposed FSS is measured by the space method in the anechoic chamber. As shown in Figure 5, the network analyzer (PNA, Keysight N5247A) is connected to the standard horn antenna at both ends. The two antennas are placed opposite each other horizontally, and a 60 × 60 cm^2^ copper plate is placed in the middle. The center of the copper plate has a hollow area of 20 × 20 cm^2^ where the FSS under test (FUT) is placed, and the rest of the area is filled with absorbing materials. The transmission coefficient of FUT is obtained by calculating the difference of the |S_21_| with FUT and without FUT. As shown in Figure 6, the measured result is in good agreement with the simulated result. Furthermore, a feature selective validation (FSV) method, which is a central technique to compare different datasets [30], was applied to give a statistical comparison between the simulated and measured results. A *GRADE* value of 3 and *SPREAD* value of 2 were obtained, demonstrating the good agreement. Moreover, the proposed FSS exhibit high efficiency in suppressing the electromagnetic wave transmitting at 3.5 GHz.

Mutual coupling reduction in MIMO systems has attracted increasing attention. The proposed stopband FSS can effectively depress mutual coupling, and the specific performance will be shown in a 1 × 2 DRA array.

**Figure 7:**
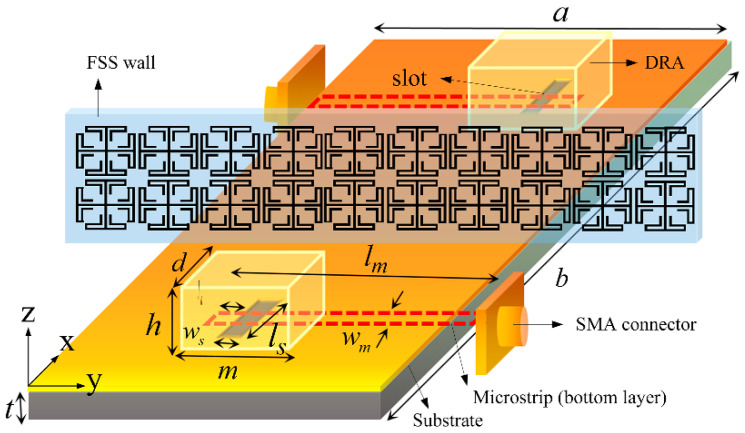
Schematic illustration of the 1 × 2 dielectric resonator antenna (DRA) multiple-input–multiple-output (MIMO) system with FSS wall. *m* = *d* = 14.5 mm, *h* = 7 mm, *a* = 35.5 mm, *w_m_* = 2.48 mm, *l_m_* = 20 mm, *l_o_* = 10 mm, *l_s_* = 13 mm, *w_s_* = 2 mm, *t* = 1.6 mm.

**Figure 8:**
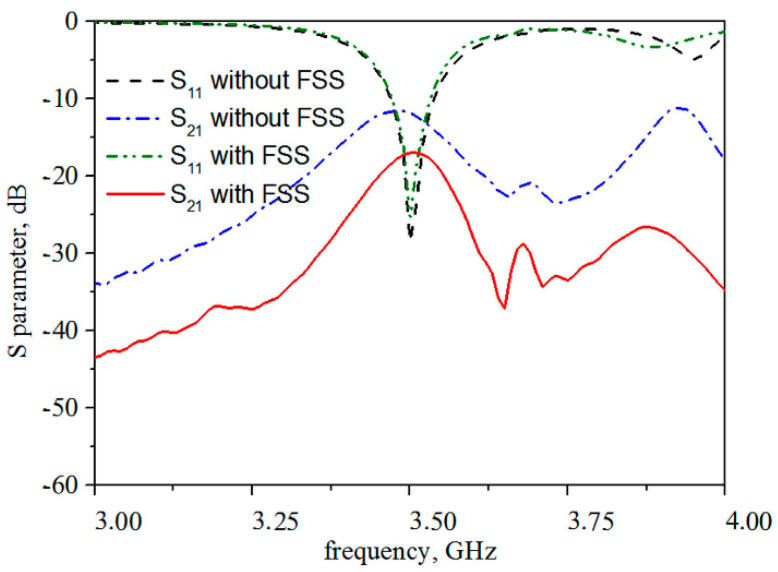
Simulation results of S-parameters for 1 × 2 DRA with and without HCGF FSS wall.

The DRAs, constructed by rectangular dielectric resonators with relative permittivity of 37, are placed on a 0.787-mm-thick Rogers 5880 substrate ($\varepsilon_{r}$ = 2.2, tanδ = 0.0009) in an arrangement of 1 × 2 array with a center-to-center distance of 41 mm corresponding to λ/2 at 3.5 GHz, as depicted in Figure 7. An FSS wall consisting of 2 × 10 unit cells along the E-plane is placed in between the two DRAs. The number of FSS unit cells is optimized by a parametric study to match the operating frequency of 3.5 GHz, thus eliminating any influence on the input impedance of the DRAs. Simulated S-parameters of the DRAs with and without FSS wall are depicted in Figure 8, showing a mutual coupling reduction of more than 8 dB on average (10 dB at 3.4 GHz, 6 dB at 3.5 GHz, 9 dB at 3.6 GHz).

**Figure 9:**
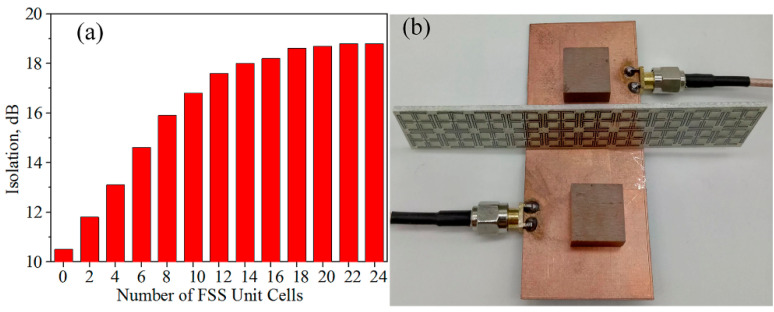
Measurement about the number of FSS unit cells. (**a**) Isolation versus the number of FSS unit cells at 3.5 GHz. (**b**) The prototype of the DRAs array.

In order to control the size of the MIMO antenna, the effect of FSS unit cell number is investigated, as depicted in Figure 9a. The more FSS unit cells there are, the greater the isolation between antenna elements is. When the number of unit cells exceeds 20, the isolation remains virtually unchanged (~18 dB). Considering the isolation and size of the antenna, the proposed FSS consists of 2 × 10 elements, as shown in Figure 9b.

**Figure 10:**
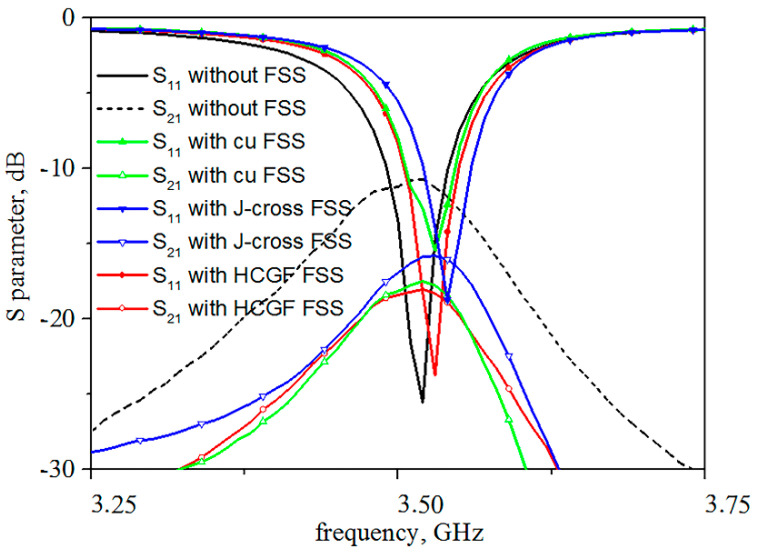
S-parameters measurement results of 1 × 2 DRA MIMO antennas with different FSSs.

A control experiment has been carried out to compare the reflection and transmission coefficient. An FSS with a J-cross structure made of graphene, two FSSs of the proposed structure with the same dimensions made of copper, and graphene are placed in the middle of the MIMO antenna array. The S-parameters measurement results of 1 × 2 DRA MIMO antennas with different FSSs are shown in Figure 10. The mutual coupling is reduced by 7.42 dB at 3.5 GHz with the proposed HCGF FSS. It can be seen from Figure 10 that all three different FSSs have a good suppressed mutual coupling effect, but the proposed FSS has better performance than the FSS with a J-cross structure. The decrease in amplitude of the proposed FSS is averagely 2 dB larger than that of the J-cross FSS around 3.5 GHz, which is in agreement with the simulation results. The proposed FSSs made of copper and HCGF have almost the same transmission coefficient, but the former has a serious effect on the reflection coefficient of the antenna system. An electromagnetic wave is more easily reflected on a metal surface, which is one of the advantages of graphene FSS.

**Figure 11:**
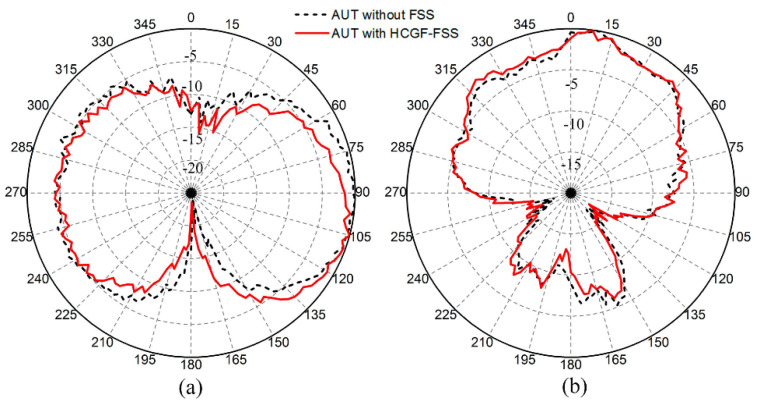
Normalized radiation patterns measured at 3.5 GHz in the x-z plane (**a**) and the y-z plane (**b**).

In addition, normalized radiation patterns of the antenna at 3.5 GHz are measured in the microwave anechoic chamber. We observed from Figure 11 that the radiation properties of the antenna are hardly influenced by the proposed FSS.

## 4. Conclusions

A novel stopband FSS made of high-conductivity graphene films to suppress the mutual coupling between two antenna elements has been designed and fabricated. The proposed FSS structure can effectively reduce the size of the unit cell by 88.89% compared with the traditional cross structure. In addition, the stopband of the proposed FSS covers the 3.5 GHz band. Demonstrated by S-parameter measurements, the mutual coupling between the DRAs has been reduced by 7.42 dB at 3.5 GHz. In conjunction with the radiation pattern measurements, the proposed FSS proved excellent isolation efficiency without compromising the performances of DRA antennas at the operating frequency. Thus, the fabricated FSS can serve as a good candidate for reducing mutual coupling in the MIMO antenna system for 5G communication.

## Data Availability

Not applicable.
